# A novel, low cost, and accessible method for rapid fabrication of the modifiable microfluidic devices

**DOI:** 10.1038/s41598-020-73535-w

**Published:** 2020-10-05

**Authors:** Mohsen Annabestani, Pouria Esmaeili-Dokht, Mehdi Fardmanesh

**Affiliations:** Department of Electrical Engineering, Sharif University of Technology, Tehran, Iran

**Keywords:** Biomedical engineering, Mechanical engineering, Lab-on-a-chip

## Abstract

As microfluidic chips are evolving to become a significant analysis tool toward POCT devices, it is crucial to make the cost and the time required for the fabrication process of these chips as low as possible. Because of the multidisciplinary nature of these systems and the collaboration of many different laboratories and organizations from vastly various fields with unequal types of equipment, it is essential to develop new techniques and materials to make the integration of disparate systems together more straightforward, accessible, and economical. In this paper, we present ethylene–vinyl acetate (EVA) as a new polymer-based material for the fabrication of different microfluidic chips, which brings new features and tools in fabrication, integration, and functionality of microfluidic systems. We put this material next to PDMS for comparison between various aspects of these materials. We have shown that besides the low-cost ability, ubiquitousness, geometrical modifiability, and ease of fabrication of EVA chips, due the lower hydrophobicity and lower terahertz (THz) absorption of EVA than PDMS, EVA chips, in comparison to PDMS counterparts, can work faster, have less number of channel blocking and can be used in THz biosensing application like metamaterial-based cancer detection. Finally, several devices are made using EVA to demonstrate the functionality and versatility of this material for the fabrication of microfluidic chips.

## Introduction

Microfluidic as an emerging technology has tremendous potential to become one of the essential parts of everyday life and already is of substantial interest in a wide variety of different fields like chemistry, biology, physics, biomedical engineering, etc^1–8^. As this technology moves forward, its potential is unveiling, showing great promises for integrating into multidisciplinary systems^9^. To pave the path for improvement and integration within different laboratories with different equipment, it is essential to bring the cost of these systems as low as possible and make them easier to fabricate, modify, and integrate. Fabrication of microfluidic chips with PDMS consists of first producing mold and then pouring the PDMS mixture into the mold, put it in the desiccator to get rid of the bubbles, curing it at a specified temperature and period, and finally plasma bonding of it on glass substrate^10^. Although it is a standard method for the fabrication of microfluidic chips, it is still time-consuming and costly in some countries with less access to this material and required instruments of photolithography.

PDMS has excellent features like flexibility, transparency, biocompatibility, and gas penetration^11^. However, its low features like weak transmission behaviour in the IR range, long cure time, and the fact that after the chip is cured, it cannot be modified are some of its drawbacks^12,13^. In this paper, we introduced ethylene vinyl acetate (EVA) as an alternative for PDMS, preserving most of its great features and adding some more. In the rest of this paper, we will demonstrate the process which is used to fabricate and modify EVA based microfluidic chips and different method for bonding them^14^.

## EVA-based microfluidic chips

**Figure 1:**
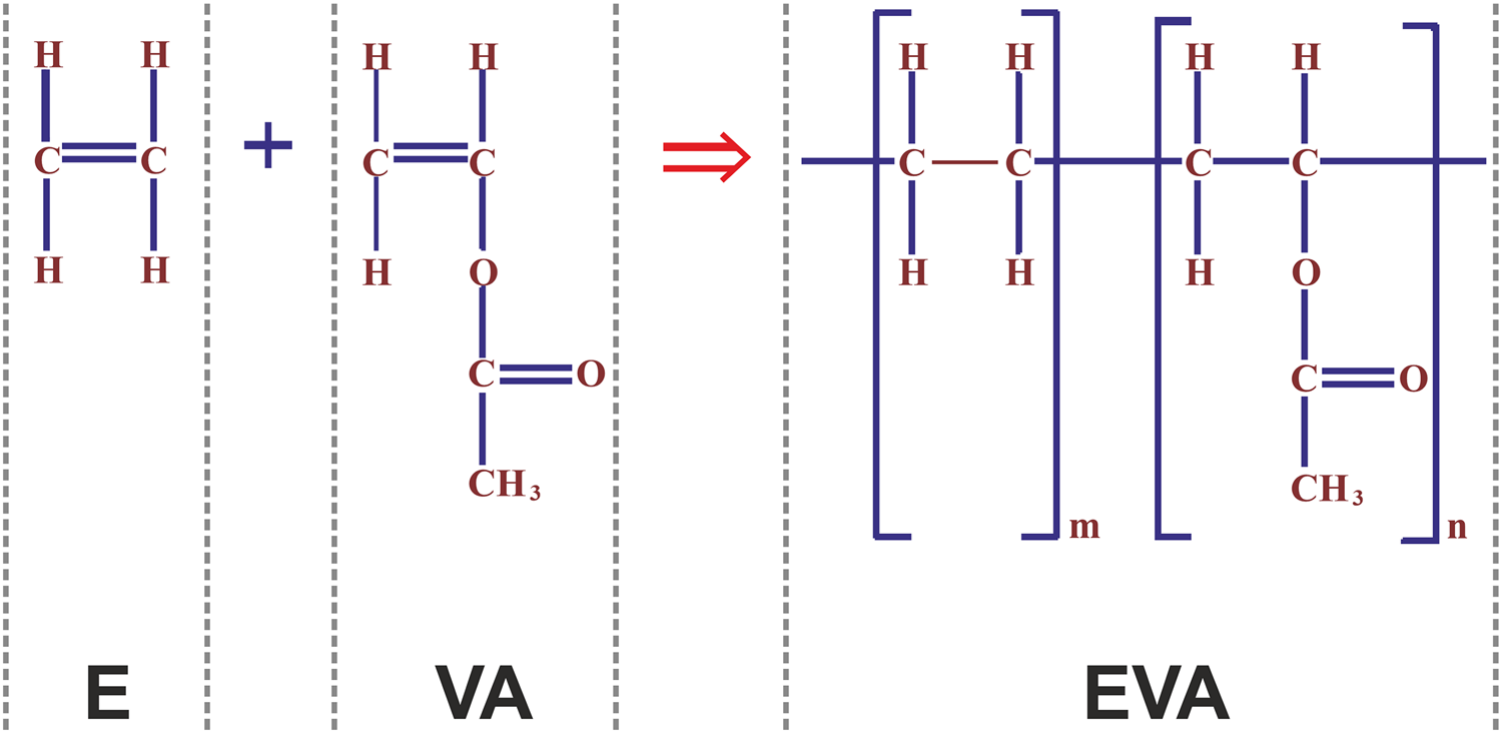
Ethylene–vinyl acetate copolymer chemical structure.

Ethylene-vinyl acetate (EVA), also known as poly (ethylene-vinyl acetate) (PEVA), is the copolymer of ethylene and vinyl acetate (Fig. 1). The weight percent vinyl acetate usually varies from 10 to 40%, with the remainder being ethylene^15^. The materials with approximately 11% VA are used as hot melt adhesives, which we are talking about here. It is affordable, accessible, easy to use, biocompatible, semi-flexible, and has good transparency in visual and IR range^15,16^. As for the pricing, for a fair amount of 3 g of the material for each typical microfluidic chip, PDMS will cost around 1 US dollar while EVA has a mere cost of 0.6 cents for each chip and also the higher thermal cost and time-consuming process of curing and bonding of PDMS adds to the cost more than that. Beside the above mentioned features, one of the most beneficial properties which give this material its distinctive attention is the ability to modify the geometry and structure of the chip or integrate different active systems into it even after the chip is bonded. In continue of this part, the fabrication procedure of EVA-based chips will be described and their features we will be explained in the next part.

### Fabrication

**Figure 2:**
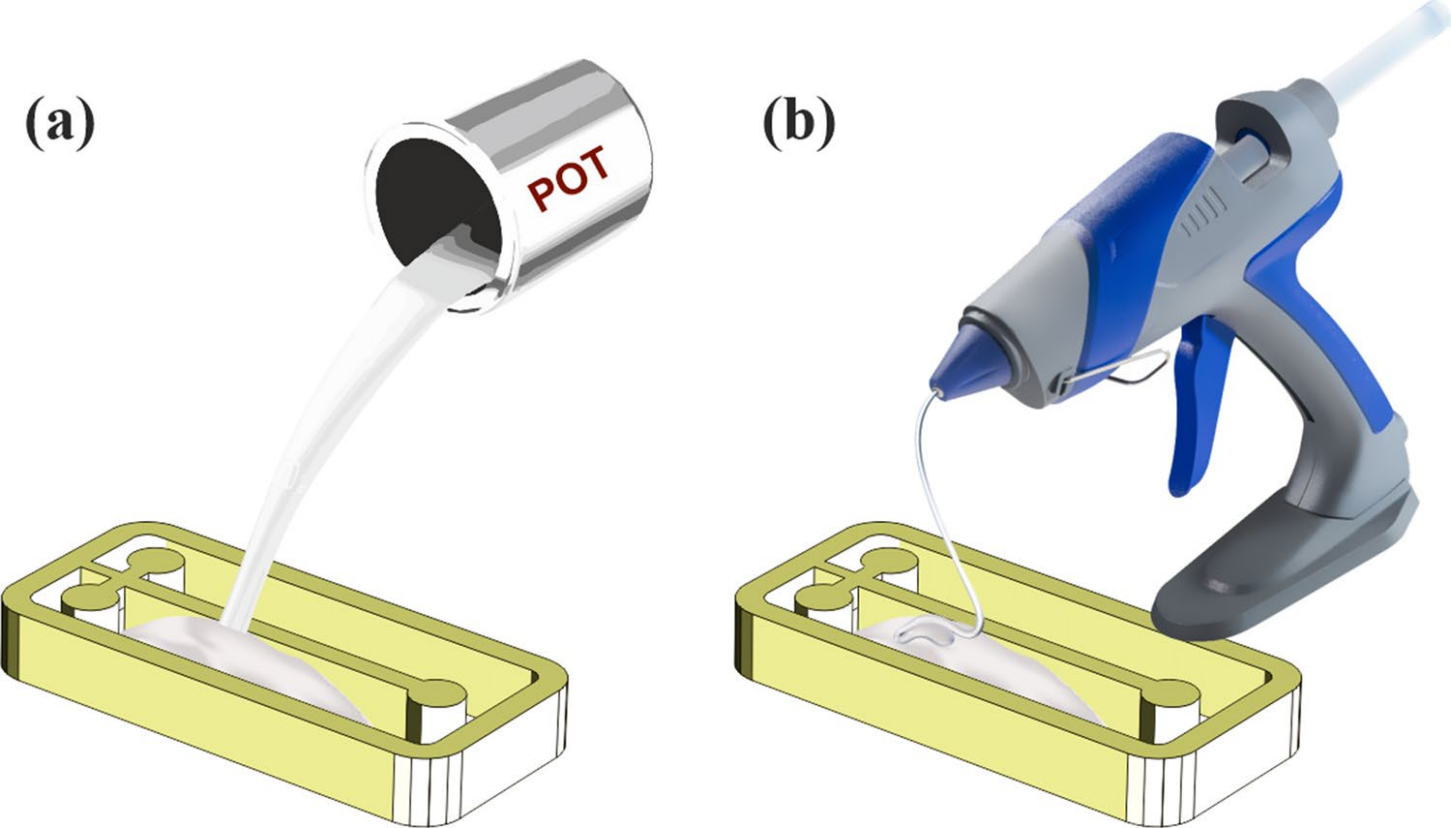
Melting of the EVA: (**a**) using pot, (**b**) using the gun directly.

Chip fabrication using this material is fast and straightforward. At first, a mold is prepared either by 3D printing or photoresist and lithography or other conventional methods^17^. After that, melted hot glue (EVA11%) should be used as filling material for the mold. It can be done either by a pot to first melting EVA and then pouring it into the mold or only using the hot gun itself to fill it (Fig. 2). The first method is a little time consuming but offers better results as the bubbles go out in the melting process, and we have a uniform melted EVA to fabricate chips. This step is done in 150° for 15–20 min. As for the second method, it is faster but requires more effort in the next stage of fabrication to result in a bubble-free chip.

**Figure 3:**
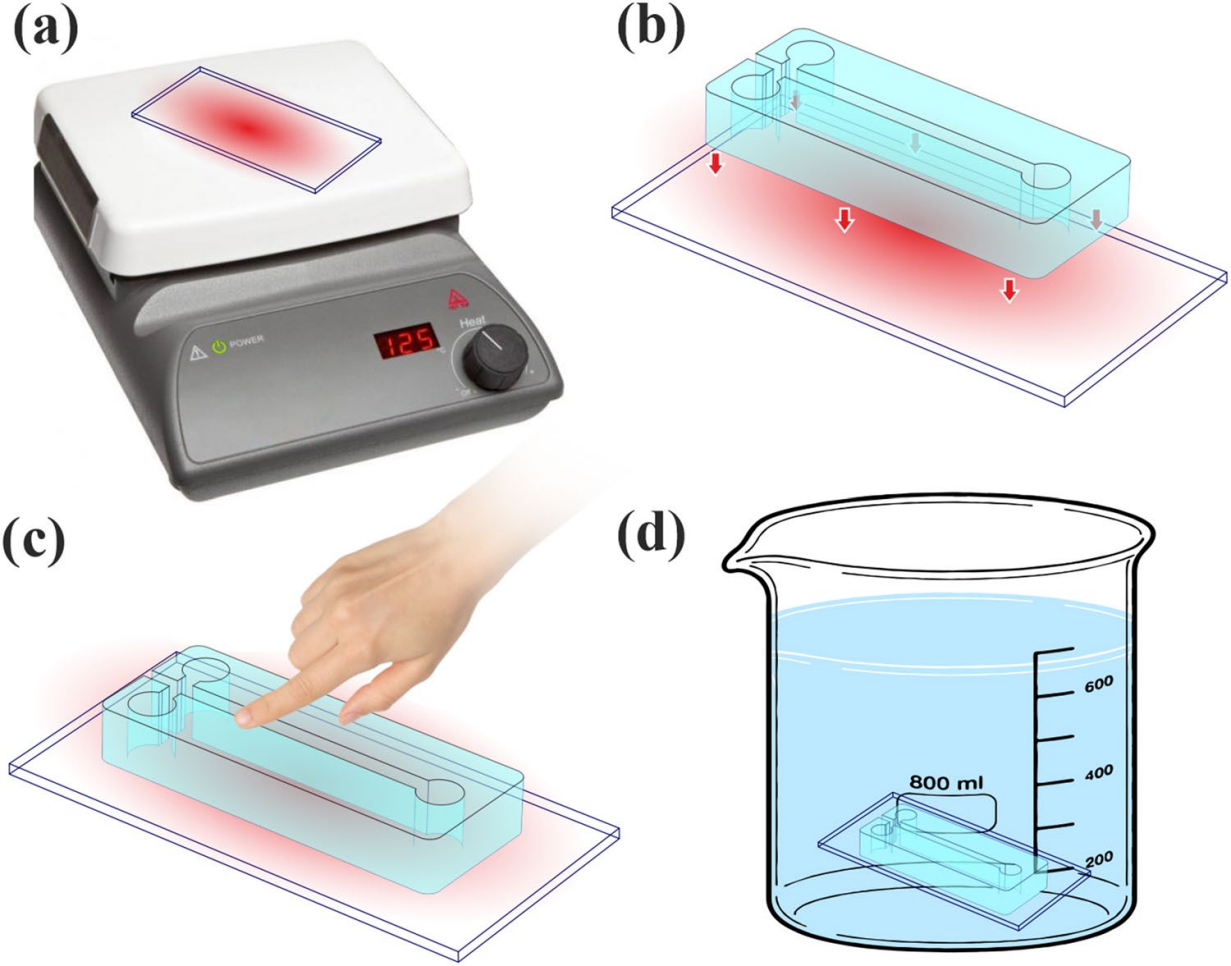
Bonding the EVA chip using a heater and cold water. (**a**) Heating the piece of glass on the heater, (**b**) placing the fabricated chip on glass, (**c**) putting pressure for some time, which depends on the thickness and channel dimensions, and (**d**) submerging into the water quickly after the last step.

In order to prevent the EVA from excessive adhesively to the mold, a pure lubricant can be used so it can be peeled off easily from the heat-sensitive materials like PLA or ABS mold. Then inlets and outlets are pierced through this side of the chip. Finally, to complete the chip, it needs to be bonded with a piece of glass. Usually, bonding the chip directly to the glass will produce desired results; however, to sustain more pressure in channels, the other side of the channel can be coated with a uniform sheet of EVA because of the more adhesivity of EVA with itself. The thermal bonding of the chip (both EVA-EVA and EVA-glass) can be done by two techniques. In the first method (Fig. 3), a piece of glass is placed on a heater and heated to 120°. Then the top layer is placed on it with minimum push (like finger touch) to make contact with the glass or coating of the EVA on the glass. Finally, after 10 s or less, which depends on the thickness and channel dimensions, the chip will be entirely submerged into cold water. In this part, the amount of pressure that is needed for bonding should not deform the melted layer on the glass to ensure the channels are open, and the submerging step should be done as quickly as possible to result in a defect-free chip. In the second method for a more reproducible and defect-free result, the thermal bonding can be done by using a microwave oven. In this step, a piece of glass is placed in the microwave. Then water is sprayed on it so that a uniform coat of water droplets will be created on the surface. Finally, the top side of the chip is placed on the glass, and the microwave will run for around 10 s. The amount of time that is needed for this step is dependent on the weight of the top layer, and the power of the microwave, which is used so try and error is part of the process to find the desired results.

## EVA properties for microfluidics

To have practical microfluidic devices, we need to choose appropriate materials that can satisfy the required conditions of microfluidic applications. These requirements are dependent on the application; for example, in an optofluidic system, it is necessary to use a transparent material, or in continuous and permanent devices, we need to use some materials with a low level of hydrophobicity to immune the system against channel blocking. Hence, in this part, we want to investigate some critical features of EVA (in comparison to PDMS) that we may need them in microfluidic devices. More specifically, biocompatibility, flexibility, transparency, absorption, and hydrophobicity are the features that are investigated for EVA.

### Bio compatibility

One of the most significant features of materials like PDMS is biocompatibility for cell viability and in-vitro cell culture. As it is stated in different reports, EVA has demonstrated a long and successful role in a variety of medical applications and has been an innovative material in those applications^18,19^. Among some of them, we can name the use in parenteral delivery of blood, parenteral delivery of compounded fluids, parenteral delivery of biologics, and medical bags suitable for cryogenic stem cell storage. So as it is widely used in food and drug delivery industries in different compounds and structures it is clear that EVA is biocompatible and offers the same usability for a vast variety of biosystems^18–25^.

### Flexibility

**Figure 4:**
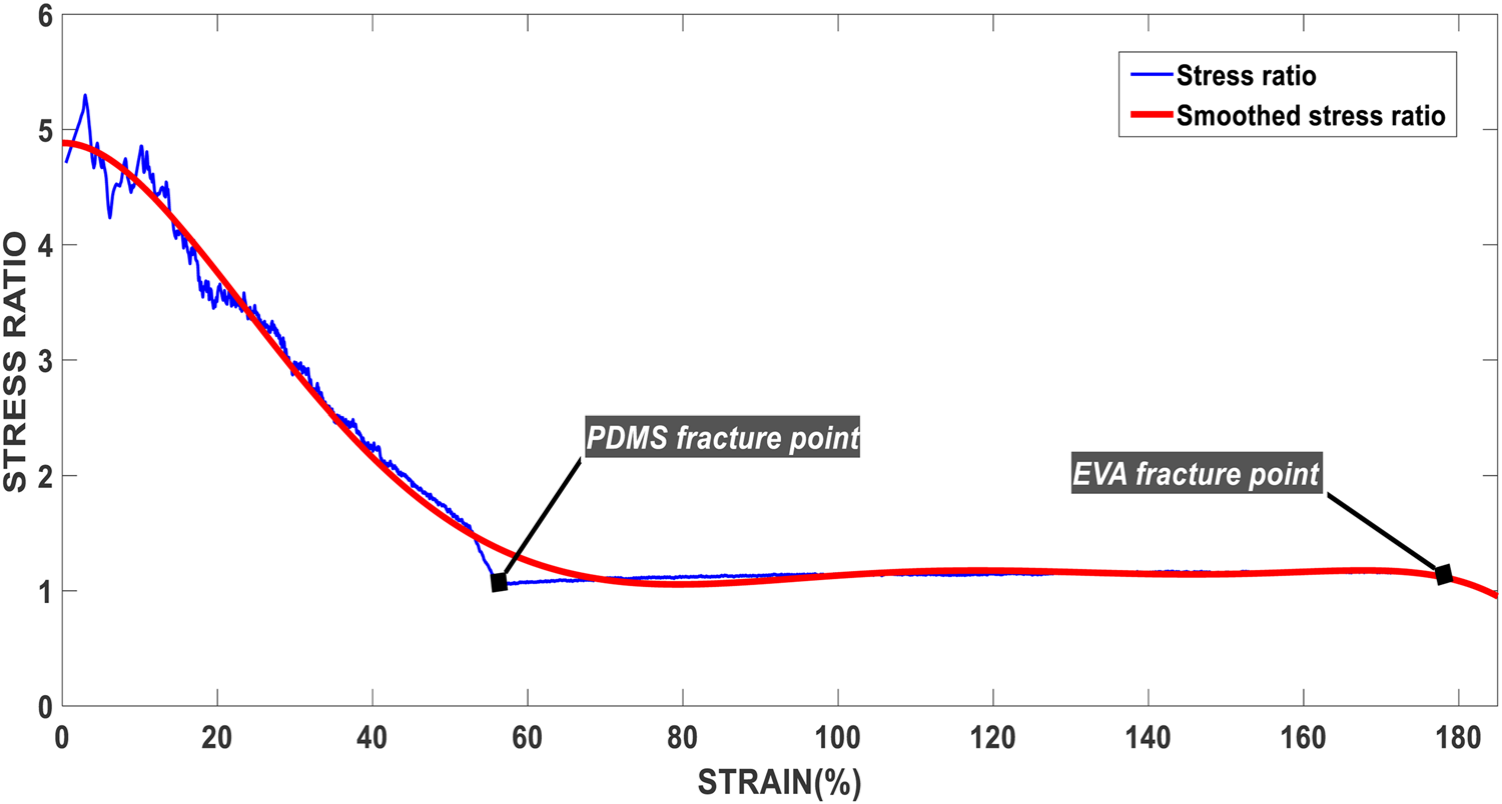
Strain–stress diagram of the ratio of EVA to PDMS ($\frac{EVA}{PDMS}$).

The strain–stress ratio diagram of PDMS and EVA is shown in (Fig. 4). In this experiment we’ve added strain to both identical samples and measured the stress. As Fig. 4 shows, it is evident that EVA withstands more stress for the same amount of strain, which means it is less elastic. PDMS reaches its fracture point at 55% strain, and EVA continues its non-elastic behaviour till 180% strain, which states the fact that EVA is more robust but shows less flexibility in different situations. However, in thin enough microfluidic chips (less than 5 mm), which is adequate for most of the requirements, there is a slight difference in terms of flexibility, and both can be considered flexible. For a more thorough and wide investigation of the mechanical properties of EVA and other thermoplastic materials and a side by side investigation of PDMS and EVA together, we refer to some of the references^26–28^.

### Terahertz transparency and absorption

**Figure 5:**
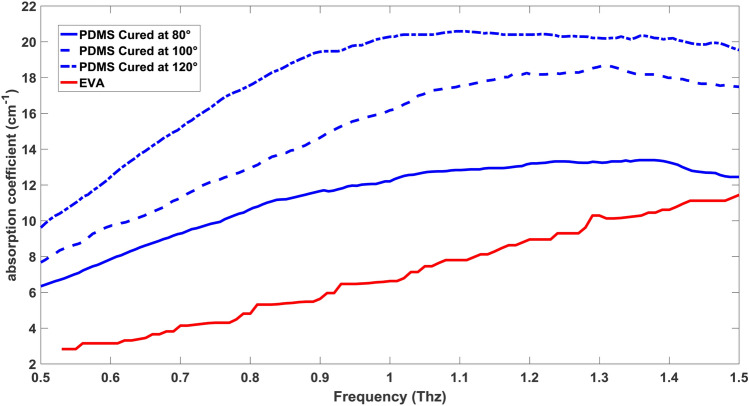
Comparison of absorption coefficient of PDMS at different cure temperature with EVA(12%).

**Figure 6:**
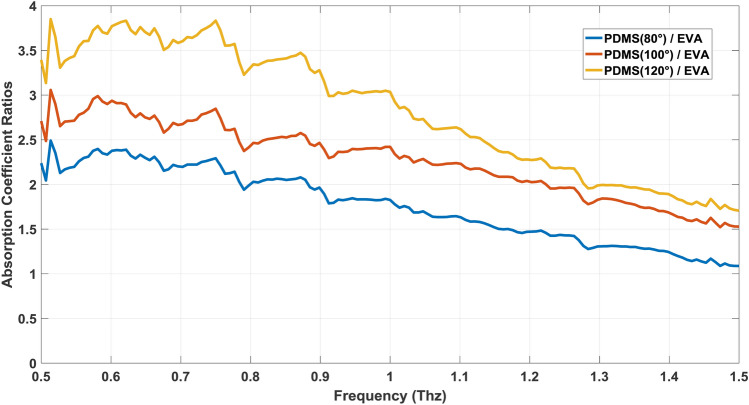
The ratio of the absorption coefficient of PDMS at different temperatures and EVA.

In terms of transparency in different frequencies, complete research was done independently for these two materials in the terahertz spectrum, which we will use the results for comparison between them^13,29^. As it was stated, melting adhesive has 11% of vinyl-acetate that it is very close to the 12% that we can find in literature, and so the comparison is precise and trusted enough^29^. As for the EVA, we have absorbance data instead of the absorption coefficient, so by using the thickness of the sample, which is 1 mm, the corresponding absorption coefficients should be calculated using the below equation derived from Beer–Lambert law:

1 $$absorption coefficient=\mathrm{ln}\left(10\right) \times \frac{A}{t}$$

where in (Eq. 1) “*A*” is absorbance and “*t*” is thickness. By using empirical data that were deduced from literature, we can compare the absorption coefficient of PDMS in different cure temperatures with EVA^13,29^. It is clear from (Fig. 5) that for the frequency range of 0.5–1.5 Thz, EVA has a lower absorption coefficient compared to PDMS in different cure temperatures. For a better understanding of the comparison, a graph of the ratios of absorption coefficients is shown in (Fig. 6) which shows clearly even at high frequencies around 1.5 Thz, EVA has lower absorption, and the ratio is still over one. Although the absorption coefficient of PDMS (80°) and EVA (12%) get closer at 1.5 Thz, chip fabrication with PDMS is more time-consuming at this temperature, which increases the cost of production for this material. So, we can see that EVA has the upper hand in terms of transparency in the Terahertz region, hence this material is more useful for special applications such as metamaterial biosensors, trapping, and sensing of microparticles, nano-fluidics for sensing, and so on^30–32^.

### Hydrophobicity

**Figure 7:**
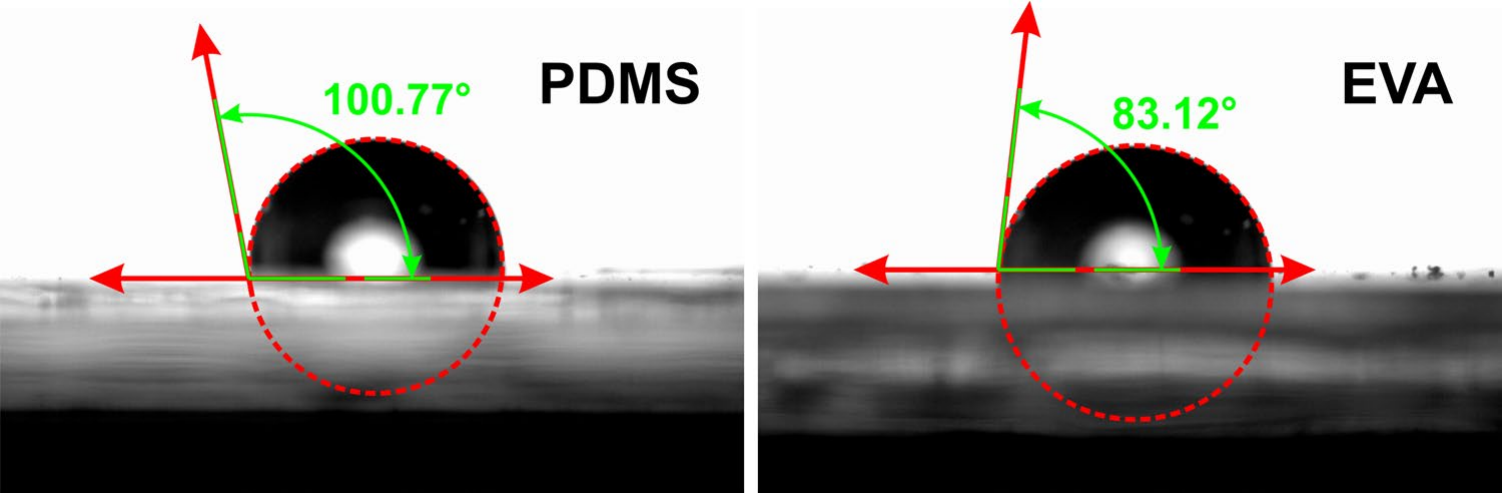
Contact angle measurement for left: PDMS and right: EVA. EVA has smaller contact angle which it means that EVA is a more hydrophilic (more wettable) and so less hydrophobic than PDMS.

In order to make a comparison of hydrophobicity of these two materials, we have measured the contact angle of DI water droplet in both materials as depicted in (Fig. 7). By measuring this parameter, it was concluded that EVA has smaller contact angle which it means that EVA is a more hydrophilic (more wettable) and so less hydrophobic than PDMS that it is an essential feature for microfluidic devices. Because in highly hydrophobic material like PDMS, the microchannels will be blocked after a couple of uses, but in less hydrophobic material like EVA, we will face the blocked channel very seldom, and so we can use the EVA microfluidic chips for several times without any problem. The experiment has been done by a commercial optical contact angle measuring and contour analysis systems (OCA-DataPhysics Instruments) and has been repeated for several points on the EVA and PDMS surfaces and the averages has been reported here (Fig. 7).

## Experimental validation

To test the processes that were explained earlier and to show the functionality of this material, three different passive, and active chips were fabricated using this material. First, a passive spiral shape sorter was fabricated using a SU-8 mold and bonded to a piece of glass using a microwave. Second, in order to show the advantage of this material compared to PDMS for integration and post-modification, an electro-active polymer based active microfluidic mixer was designed and fabricated. And finally, a maze mixer with three inlets was fabricated using the same method and tested using two different colours.

### Spiral mixer/sorter using SU-8 mold

**Figure 8:**
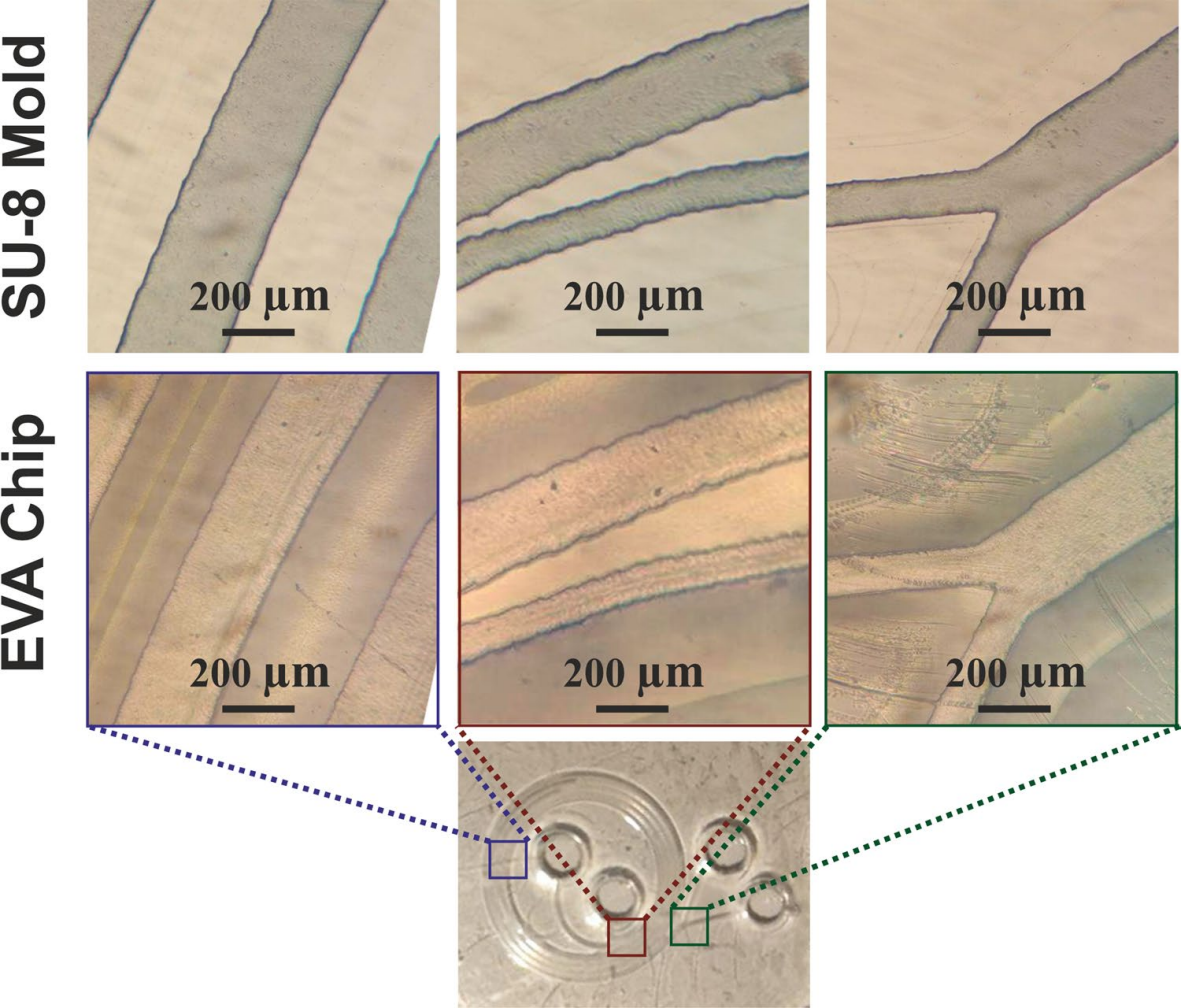
A typical spiral mixer/sorter with EVA, (up): SU-8 mold in three locations of the chip, (middle): fabricated EVA chip in the same three locations. (Down): the whole of fabricated EVA chip.

As (Fig. 8) shows, an EVA spiral chip was fabricated using SU-8 mold on the sio2 substrate. It is clearly demonstrated that all the details are entirely identical to the mask and EVA fabricated microfluidic chip. It can guaranty that EVA can make microfluidic chips as precise as the chips that we can make by standard SU-8 based soft lithography procedures besides that it is extremely cheaper, modifiable and has a lower probability of channel blocking.

### Active mixer

**Figure 9:**
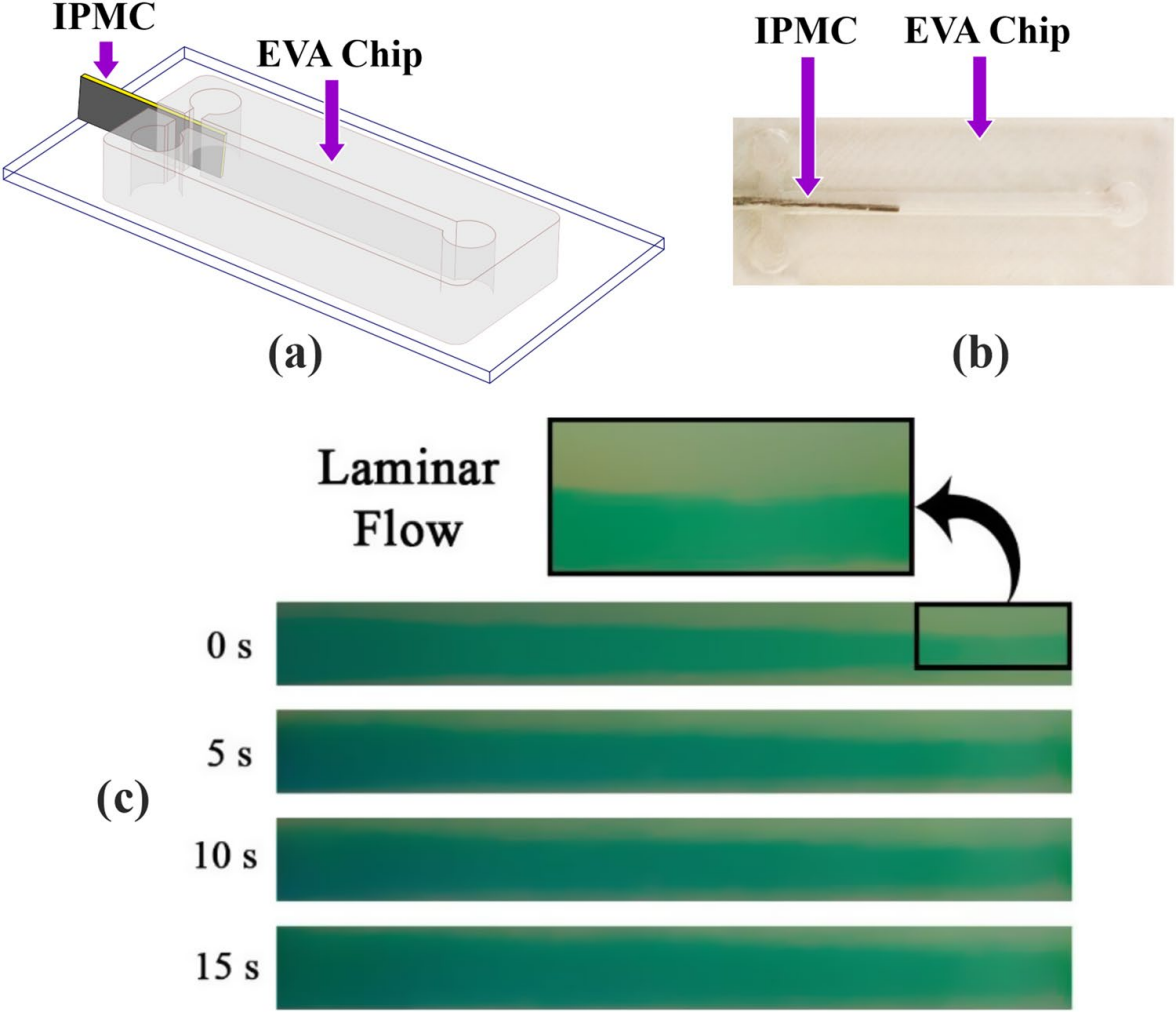
An electroactive polymer-based active micro-mixer fabricated by EVA. (**a**) 3D schematics, (**b**) fabricated version, and (**c**) its mixing performance test.

**Figure 10:**
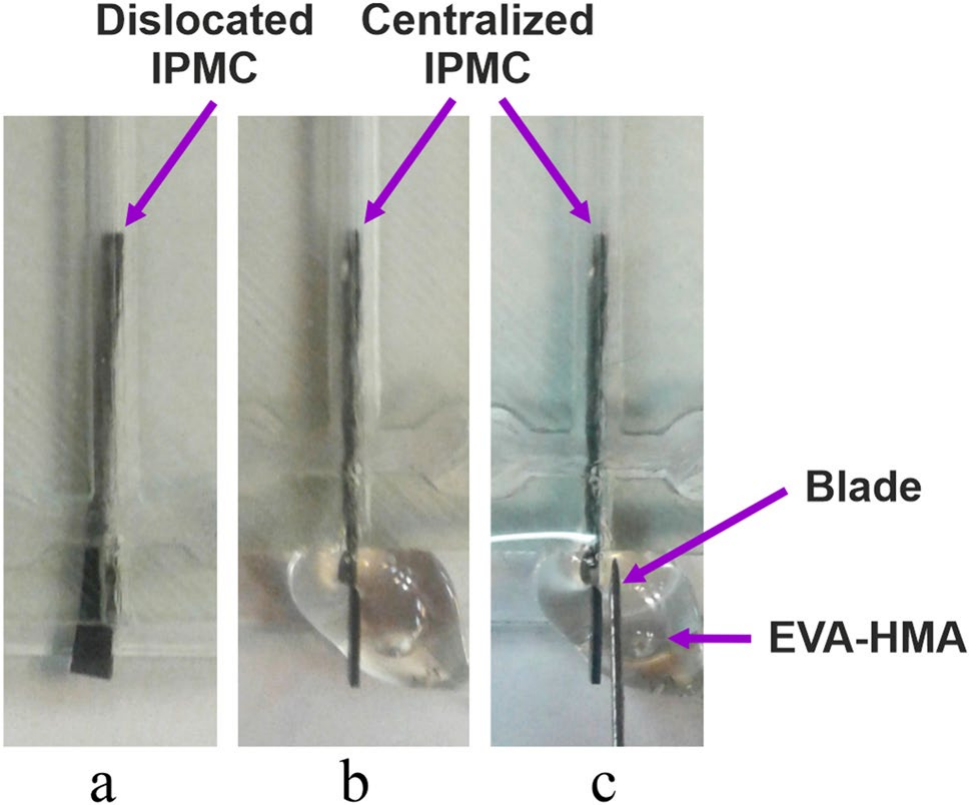
Fixing and centralization of the location of IPMC actuator into the an EVA channel. (**a**) Dislocated IPMC, (**b**) fixing the IPMC in the center of the channel using a droplet of HMA, (**c**) remove the unwanted HMAs using a blade.

In our previous work, we designed and fabricated a PDMS based active micromixer that was equipped with IPMC electroactive polymer^33–36^. Now, we have fabricated the same chip but using EVA as base material instead of PDMS (Fig. 9). In the process of embedding IPMC into the channel, the biggest problem was balancing the IPMC location and sealing of the channel. To solve these issues, first, we should make sure that IPMC is located in the center of the channel and has no friction to the channel walls, and second, the interface of the channel and the body of the IPMC should be fully sealed. Solving these two challenges are very difficult and tricky when we want to use PDMS chips; for example cure time of PDMS is around two hours in 85° that; this curing procedure has two critical problems; first IPMC actuator as an Ionic actuator will be semi-dehydrated in 85° and cannot work correctly, and second, the PDMS is a liquid, and when we want to seal the interface of IPMC and the channel wall it may penetrate the channel and so blocks the channel, hence the sealing procedure in PDMS chip is very tricky and may we lose some chips to make the proper one. In EVA chips but by merely putting the IPMC into the channel and locally heating the interface of the IPMC and the wall, we can seal the channel complete and fast enough. As to fine-tuning the IPMC location in the channel every time, we only need to make a change, by local heating the interface, and moved the IPMC quickly, while, in the PDMS chip, we have to fabricate the whole chip again in case of dislocation of the IPMC in the channel. As it is shown in the (Fig. 10a), by placing IPMC in the microfluidic channel, the micromixer cannot sustain its straight shape and tilt to one of its sides. To fix the IPMC in the middle of the channel, one drop of the EVA is added to the input interface, while the IPMC is held in place manually (Fig. 10b). This whole process took about 30 s, and compared to PDMS, is substantially faster and more risk-free from channel getting blocked due to the PDMS leakage into the channel before curing. After EVA entirely seals the interface, the remaining EVA around the interface is cleaned easily using simple tools like blade, soldering device etc. This active mixer is only one of the examples that demonstrates how easier it is to integrate different systems into the EVA microfluidic chips in comparison to conventional PDMS ones and we can find verity of other embedded active microfluidic chips as examples.

### 3D printed mold-based Maze shaped micromixer

**Figure 11:**
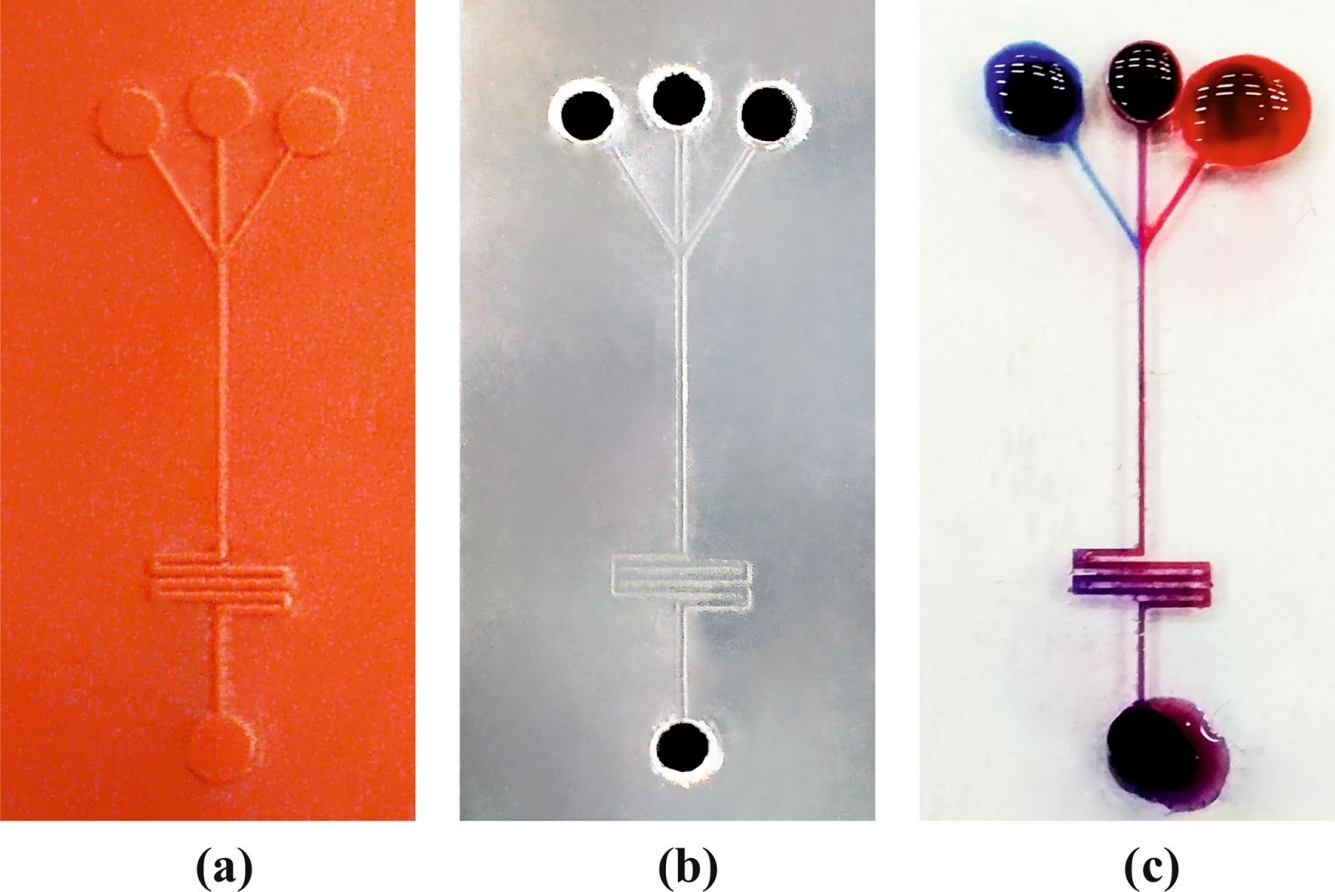
(**a**) 3D printed mold by DLP method and casting resin. (**b**) Fabricated chip by EVA, (**c**) fabricated chip under the test.

Another method that we can use to make the EVA chips is based on 3D printed mold. For small size channels (around 100 µm) we prefer to use the digital light projector (DLP) method and print the molds using casting resin. However, FDM and SLA printing methods are also applicable for making EVA molds but based on our experiment, DLP methods and printing the casting resin have better results. Here to show the feasibility of the EVA chip fabrications using 3D printing molds, we have fabricated a simple maze shaped 200 µm channel micromixer (Fig. 11) with three inlets and bonded to the glass substrate. To show the functionality of this chip, we tested it by three dyes in the inlets, and as it has been shown in (Fig. 11c), it works appropriately and even faster than its PDMS counterpart.

## Conclusion

In this paper, we introduced EVA as a new alternative to PDMS for the fabrication of different microfluidic devices. We showed that this material possesses superiority in terms of economic cost, fabrication time loss, handling, inerrability, absorption, bonding, and ease of use. Both materials' production cost was evaluated in terms of the material cost, which showed great promises for the mass production of POCT and healthcare devices based on EVA microfluidic chips, and the ease of production and processing of EVA showed a considerable leap toward the commercializing POCT devices based on microfluidics. The mechanical behaviour of these two materials was investigated in which EVA showed less flexibility compared to PDMS, although sustaining more strain compare to PDMS. In terms of biocompatibility, EVA has demonstrated a long and successful role in a variety of medical applications that are applicable in this matter. Furthermore, it was demonstrated that EVA is less hydrophobic than PDMS and fluids can flow faster and easier into the microchannels with lower possibility of channel blocking. And finally, we showed that the more transparent behaviour of this material in the terahertz range allows new ideas to emerge in this field as a sensing and diagnostic device for cancer and different cell biology fields, which is limited by the transparency of the PDMS in the terahertz region.
